# Supplementary material for: MetaRibo-Seq measures translation in microbiomes
Source: Nat Commun. 2020 Jun 29;11:3268. doi: 10.1038/s41467-020-17081-z (PMC7324362; doi:10.1038/s41467-020-17081-z)
Supplement: Supplementary file 10 — Supplementary Data 7 [file 41467_2020_17081_MOESM10_ESM.zip › File2/Confidence_High_Taxonomy/3496_out.krona.html]

Javascript must be enabled to view this page.

members
magnitude
magnitudeUnassigned
count
unassigned
taxon
rank

3496\_out

6

6
2
superkingdom

phylum
1239
6

6
class
186801

186802
order
6

genus
1392389
6

6

SRS013965\_contig\_number\_36054SRS075984\_contig\_number\_20387SRS076804\_contig\_number\_13105SRS104311\_contig\_number\_44303SRS104636\_contig\_number\_25775SRS142542\_contig\_number\_contig-100\_655.84986
species
1297617
